# Comparison of Methods in the Serologic Diagnosis of Cystic Echinococcosis

**DOI:** 10.1007/s11686-024-00840-z

**Published:** 2024-03-29

**Authors:** Sidre Erganis, Fakhriddin Sarzhanov, Funda Doğruman Al, Kayhan Cağlar

**Affiliations:** 1 Department of Medical Microbiology, School of Medicine, Gazi University, 06490 Ankara, Türkiye; 2 Division of Medical Parasitology, Department of Medical Microbiology, School of Medicine, Gazi University, 06490 Ankara, Türkiye; 3 Faculty of Medicine, Akhmet Yassawi International Kazakh-Turkish University, 161200 Turkestan, Kazakhstan

**Keywords:** Anti-Echinococcus IgG, Cystic echinococcosis, Hydatid cyst, Serologic diagnosis

## Abstract

**Purpose:**

Cystic echinococcosis (CE) is caused by the larval form of *Echinococcus granulosus.* Clinical, radiologic, pathologic, and serologic findings should be evaluated together for the diagnosis of CE. The sensitivity and specificity oalf serologic tests may vary depending on the method used. In this study, we aimed to detect IgG antibodies specific to *E. granulosus* using indirect hemagglutination assay (IHA), enzyme-linked immunosorbent assay (ELISA), indirect fluorescent antibodies (IFA) and western blot (WB) tests.

**Methods:**

In our study, the serum samples of 74 patients sent to our laboratory with suspicion of CE were studied using two different commercial IHA tests, ELISA, IFA and WB test. The test results were evaluated along with radiological findings and histopathological examinations, the latter being the gold standard.

**Results:**

Of all the patients, 51 (69%) were female and 23 (31%) were male. There was a statistically significant difference between males and females (χ^2^ = 9.7, *p* = 0.002). Out of 74 patients, positivity rates for Siemens IHA, Fumouze IHA, ELISA, IFA and WB test were positive as 33 (44.6%), 35 (47.3%), 43 (58.1%), 42 (56.7%) and 38 (51.3%), respectively. The sensitivity and specificity of the tests were as follows: 66.67 and 2.31% for Siemens IHA; 70.83% and 96.15% for Fumouze IHA; 85.42%, and 88.46% for ELISA; 83.33% and 88.46% for IFA; 72.92% and 88.46% for WB test.

**Conclusion:**

There were statistically significant differences in between all five methods (*p* < 0,001). While the tests with the highest specificity was Fumouze IHA, the test with the highest sensitivity was the ELISA test. It was concluded that IHA and ELISA tests were more practical in practice because of their greater applicability.

## Introduction

Echinococcosis is a parasitic infection that occurs in humans and animals. The adult form of *Echinococcus granulosus sensu lato* (*s.l.*) is found in the small intestine of the definitive hosts such as dogs, wolves, and jackals [1]. *Echinococcus granulosus sensu lato* has different subtypes with different host specificities: *E. granosus sensu stricto (s.s.)* (G1-G3), *Echinococcus felidis, Echinococcus equinus, Echinococcus ortleppi*, and *Echinococcus canadensis* (G6/G7, G8, and G10) [2]. The larval form causes cystic echinococcosis (CE) that is located in the internal organs of many mammals such as sheep, goats, cattle, and humans. Intermediate hosts usually become infective by ingesting infective eggs excreted with the feces of the definitive host. It may produce a variety clinical symptoms or may be asymptomatic or show no symptoms depending on the location and size of the cyst. It is a neglected zoonosis, which is an important public health problem in many regions of the world and our country due to the socio-economic losses it causes [1, 3, 4].

Humans are defined as incidental hosts and not essential to the parasite’s life cycle. However, if the disease is left untreated, it has significant social and economic consequences, as well as serious morbidity and mortality. Estimated data on the global distribution of the disease show that CE affects 2–3 million people and there are 200,000 new cases every year [3]. According to the data of WHO, worldwide, CE causes about 19,300 deaths per year [5]. The exact number of all CE cases is difficult to estimate in Türkiye because data on the prevalence of CE in Türkiye is based on screening studies involving a small and limited number of population and cases usually reported by hospital records [6]. A study under the Heracles (Human cystic echinococcosis ReseArch in CentraL and Eastern Societies) Project investigated rural areas of Bulgaria, Romania, and Türkiye by ultrasound-based screening. In the study, abdominal CE was found in 53 (0.6%) of the total of 8618 people in villages belonging to six provinces of Türkiye. It is known that this study is the most comprehensive screening in Türkiye to date [6, 7].

It is estimated that the disease is much more common than previously thought because of the uncharacteristic clinical symptoms, very slow cyst development, and the problems in making a definitive diagnosis of the disease [4, 8]. Currently, the diagnosis of CE is mainly suggested by radiologic diagnostic methods. Ultrasonography imaging is often used because it allows staging of cysts [6]. In order to make a differential diagnosis of the cysts from other space-occupying lesions like tumors, abscesses, simple cysts, and to evaluate the recurrence after treatment accurately, the preliminary diagnosis should be supported using serological diagnostic methods such as indirect hemagglutination (IHA), immunofluorescent antibody test (IFA), enzyme immunosorbent linked assay (ELISA). The study of the genome of the parasite in recent years as a result of the advances in molecular biology has created opportunities to explore new approaches in understanding the biology, diagnosis, and treatment options of the parasite [9, 10].

In this study, it was aimed to detect *E. granulosus* specific immunoglobulin IgG antibodies in the sera of suspicious CE patients admitted to Gazi University Hospital by using various serological tests such as IHA, ELISA, IFA and WB.

## Materials and Methods

A total of 74 serum samples from the patients with CE based on the clinical and radiologic findings between June 2018 and June 2019 were submitted to the Clinical Microbiology Laboratory of Gazi University Hospital. All of the 74 serum samples were taken at the time of diagnosis. Patients diagnosed as CE by radiological imaging (such as ultrasonography, computed tomography or magnetic resonance imaging) or by histopathologically were selected in our study. Non-CE cases mentioned in our study were those that were pre-diagnosed as CE on a radiological basis but whose histopathological examination reports were not interpreted as CE.

Anti-*Echinococcus granulosus* antibodies in 74 serum samples were investigated using five different serological tests, i.e., two different commercial IHA (Siemens Cellognosts, Germany; Fumouze, France), ELISA (Euroimmun IgG, Germany), IFA (Euroimmun IgG, IgM, IgA, Germany) and WB method (Euroimmun IgG, Germany) tests. Radiology and pathology reports were used as confirmatory methods. For Siemens Cellognost IHA, titers equal to or greater than 1/256 were considered positive, and titres of 1/128 were considered as borderline. For Fumouze IHA, 1/320 and higher dilutions was accepted as positive, and titres of 1/160 were considered as borderline. In the IFA test, sera were diluted at 1/100, 1/320, and 1/1000 titers, and structures that gave green fluorescence under fluorescent microscopy matching the protoscolex morphology at titers of 1/100 and above were accepted as positive. The IHA and IFA tests are based on an *E. granulosus* antigen, whereas the ELISA was based on a purified antigen of *E. multilocularis*. The WB test, on the other hand, includes *E. granulosus* antigenic extract and synthetic antigens Em18, Em95, and EgAgB of E.granulosus. In the WB samples studied, the presence of at least one of the p7, p16/18, p21, Em18, or Em95 antigen bands were considered positive for *Echinococcus* spp. The presence of recombinant EgAgB antigen band in addition to one of the p21, p7, or p16/18 antigen bands showed *E. granulosus* seropositivity, while Em18 or Em95 bands indicated *E. multilocularis* seropositivity. All tests were performed following the recommendations of the manufacturers.

Chi-square (χ2) test and logistic regression analysis were used for statistical evaluation of the study data. The confidence interval was determined as 95% and *p* < 0.05 was considered significant. The kappa (κ) value obtained with the screening test was interpreted to determine the consistency of the tests with each other, and the sensitivity and specificity of the tests were calculated. The kappa (κ) value was evaluated as poor and insignificant agreement between 0 and 0.2, poor and moderate agreement between 0.21 and 0.4, moderate agreement between 0.41 and 0.6, good agreement between 0.61 and 0.8, and perfect harmony between 0.81 and 1 [11].

## Results

Out of the 74 patients, 51 (69%) were female and 23 (31%) were male. The average age of the patients was 45 (range, 3–86) years. When the test results were analyzed separately according to age groups and sex, there were statistically significant differences between the seropositivity rates of both female-male and age group distributions (*p* < 0.05). It was found to be significantly higher in women according to gender. According to age, it was found to be significantly higher between the ages of 19–39; It was found to be significantly lower in the 60–89 age range (Table 1).

**Table 1:** Comparison of Anti-Echinococcus IgG antibodies in CE patients confirmed by pathology and/or radiology reports by gender, age group, clinic, and location

Sex	Positive (*n* = 48)	Negative (*n* = 26)	Total	χ ^2^	p
Female	39	12	51	9.7	0.002
Male	9	14	23		
**Age**	**Positive** **(n = 48)**	**Negative** **(n = 26)**	**Total**	***χ*** ^**2**^	**P**
0–18	9	4	13	0.72	**0.36**
19–39	13	2	15	3.92	**0.04**
40–59	20	8	28	0.85	**0.36**
60–89	6	12	18	10.38	**0.001**
**Clinic**	**Positive** **(n = 48)**	**Negative** **(n = 26)**	**Total**	***χ*** ^**2**^	**P**
General Surgery	25	5	30	7.55	**0.006**
Internal Medicine	6	13	19	12.43	0.001
Pediatrics	4	4	8	0.87	0.35
Thoracic Surgery	4	1	5	0.54	0.46
Chest Diseases	2	1	3	0.004	0.95
Other clinics	7	2	9	0.75	0.39
**Location**	**Positive** **(n = 48)**	**Negative** **(n = 26)**	**Total**	***χ*** ^**2**^	**P**
Liver	37	21	58	0.14	0.71
Mixed	6	1	7	1.48	0.23
Lungs	-	1	1	1.87	0.17
Spleen	2	2	4	0.41	0.52
Other locations	3	1	4	0.19	0.66

The sera included in this study were submitted from the general surgery (*n* = 30), internal medicine (*n* = 19), pediatric health and diseases (*n* = 8), infectious diseases (*n* = 6), thoracic surgery (*n* = 5), chest diseases (*n* = 3), orthopedics (*n* = 1), neurosurgery (*n* = 1), and gynecology and obstetrics clinics (*n* = 1) departments. Considering the total cases, CE was significantly higher in the general surgery department compared with the other departments (χ^2^ = 7.55, *p* < 0.05). On the other hand, patients admitted to the internal medicine department were found less positive than those in other departments (χ^2^ = 12.43, *p* < 0.05). No significant difference was found in the other departments (Table 1).

According to the location of cysts in 74 patients, 58 cysts were located in the liver, seven in the liver and lung, four in the spleen, one in the lung, one in the ilium, one in the kneecap, one in the vertebra, and one in the adnex were reported. Although it was not statistically significant, the location of hydatid cyst was most common in the liver in our patients (χ^2^ = 0.14, *p* = 0.71) (Table 1).

Serum samples were studied using two different IHA tests, ELISA, IFA and WB. In the Siemens IHA in our study, 33 (44.6%) patients were positive with different titres, 3 (4.1%) were borderline (intermediate value), 38 (51.3%) were negative. In the Fumouze IHA, 35 (47.3%) patients were positive with different titres, 3 (4.1%) were borderline, and 36 (48.6%) were negative. In the ELISA test, 43 (58.1%) were positive, one (1.4%) were borderline, and 30 (40.5%) were negative. In the IFA test, 42 (56.7%) of them were positive, and 32 (43.3%) were negative. In the WB test, 38 (51.3%) sera were positive, 30 (40.5%) sera were negative, and six (8.2%) sera were at the borderline value (Table 2).

**Table 2:** Distribution of patient results according to the tests studied

	IHA				ELISA	IFA		WB
	SIEMENS		FUMOUZE					
	Titer	n	Titer	n		Titer	n	
Positive	1/256 1/512 1/1024 > 1/1024	4 6 14 9	1/320 1/640 1/1280 > 1/1280	13 6 9 7	43	1/100 1/320 1/1000	17 16 9	*E. granulosus* 29 *Echinococcus spp* 9
Total positive	**33**		**35**		**43**	**42**		**38**
Suspect	1/64 1/128	2 1	1/160 3		1	-		6
Total suspect	3		3		1	-		6
Negative	38		36		30	32		30
Total	74		74		74	74		74

Of the 74 sera studied, the results of 50 (67.6%) serum samples were consistent with each other in five tests. Twent-seven (36.5%) were found to be positive with all tests and 23 (31%) were found to be negative with all tests Of the 74 sera studied, the results of 50 (67.6%) serum samples were consistent with each other in five tests. In the WB of 38 positive specimens, 29 sera were identified as *E. granulosus* and the species of 9 sera could not be distinguished.

The results of 24 serum samples were inconsistent. Of the serum samples with inconsistent test results, 17 belonged to CE patients, and seven belonged to non-CE patients.

The histopathological examination reports of only 37 of the 74 patients could be accessed. Among these, 11 were identified as CE, and 26 were identified as non-CE. Out of the 11 patients whose pathology report indicated CE, eight had positive findings for all serological tests, whereas three had discrepancies in their serologic test results. Out of the 26 patients whose pathology report indicated non-CE, 19 had negative findings for all serological testing, while seven showed discrepancies in their serological test results.

**Fig. 1:**
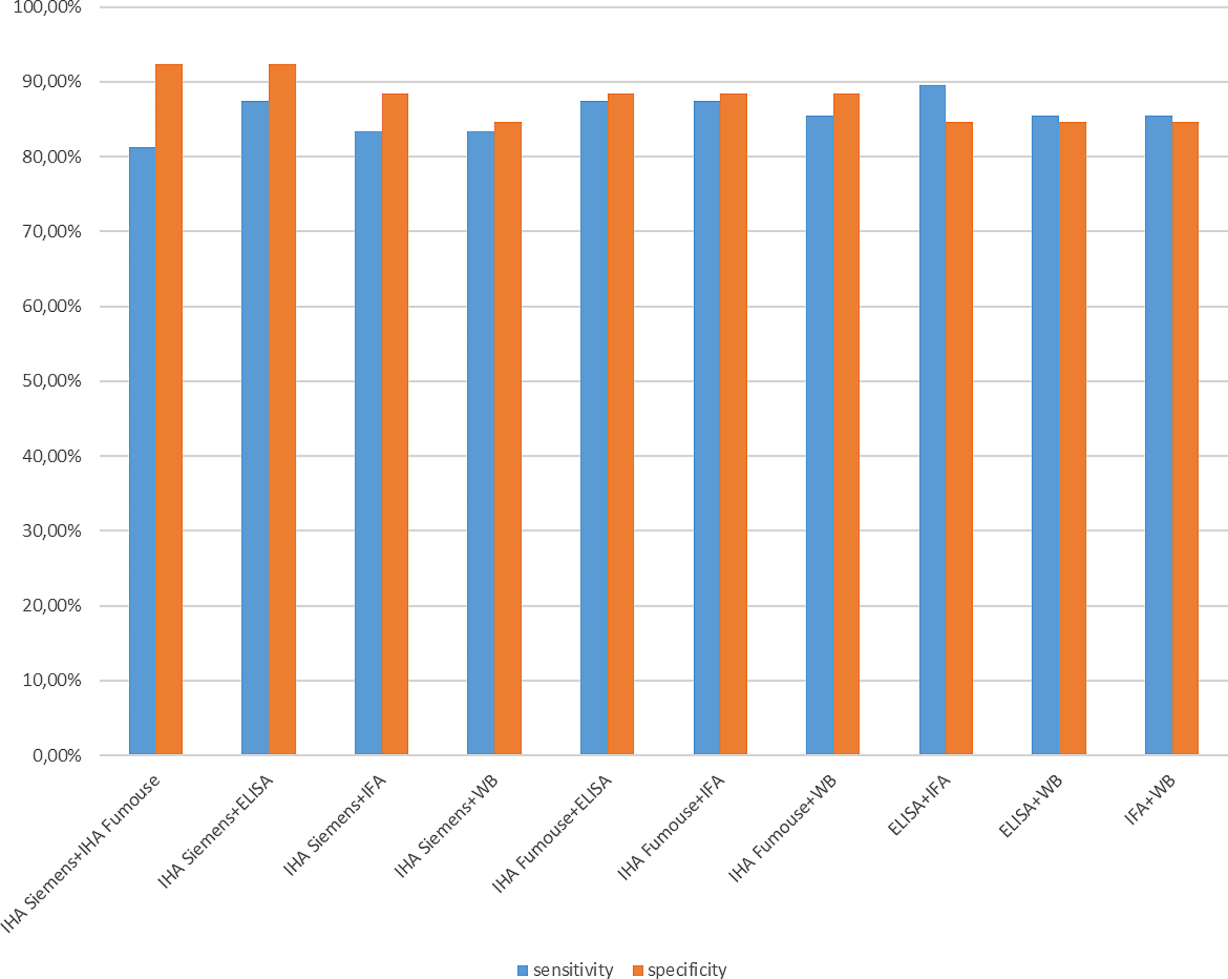
Evaluation of the diagnostic performance of the combination of two tests

**Fig. 2:**
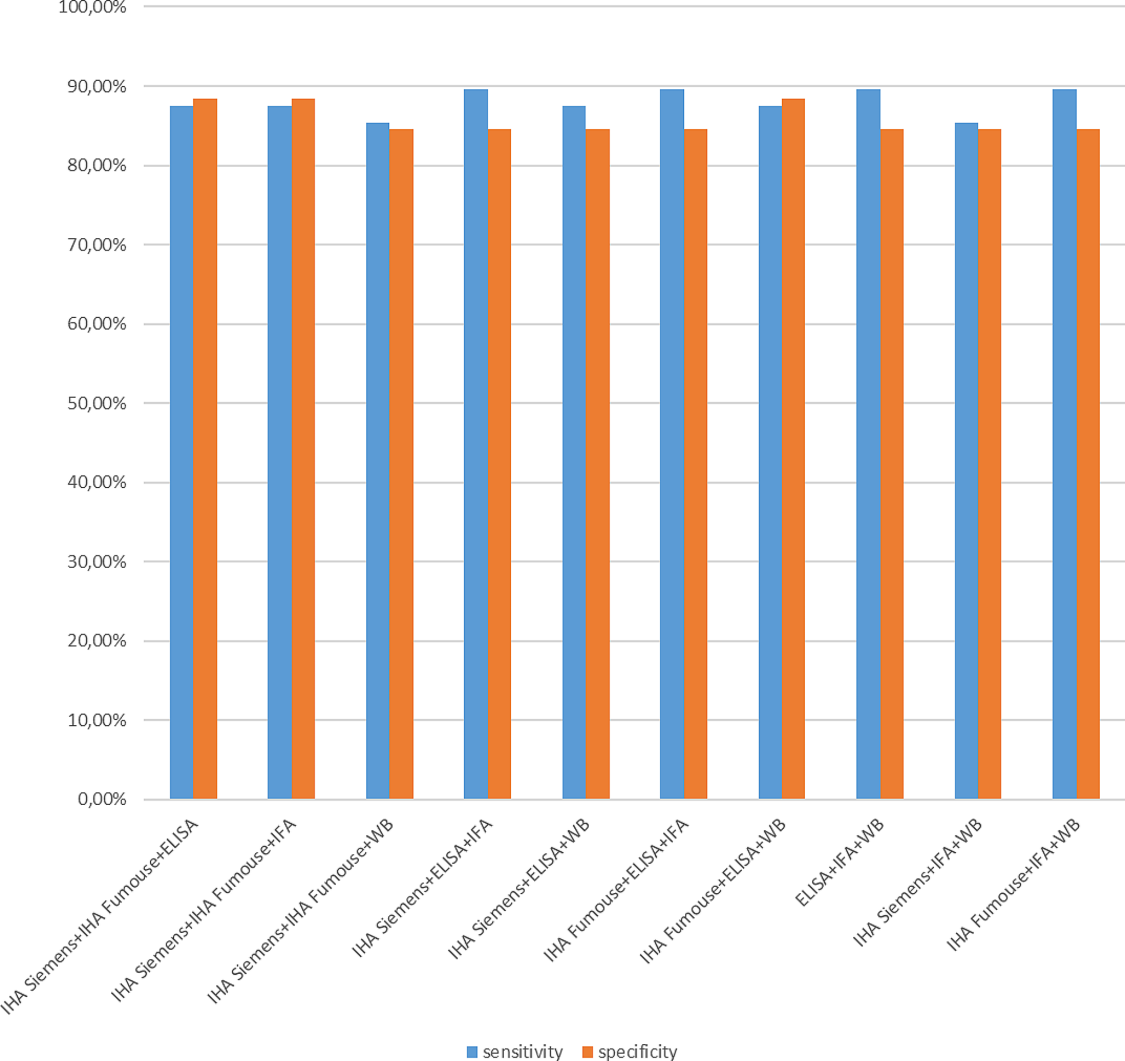
Evaluation of the diagnostic performance of the combination of three tests

We used the histopathological and radiological examination reports as the gold standard method. Borderline results were considered negative when performing statistical calculations. The results of the five methods used in our study were compared with the results of the pathology and/or radiology reports’ results, and a significant difference was found with the χ^2^ statistical analysis (*p* < 0.05) (Table 3). The sensitivity and specificity of the tests were as follows: 66.67 and 92.31% for Siemens IHA; 70.83% and 96.15% for Fumouze IHA; 85.42%, and 88.46% for ELISA; 83.33% and 88.46% for IFA; 72.92% and 88.46% for WB test (Table 4). Figures 1 and 2 shows the evaluation of the diagnostic performance of the combination of the tests.

**Table 3:** Comparison of positive and negative IHAs, ELISA, IFA and WB test results according to the pathology and radiology report’ results

		Pathology and/or Radiology		p value	*χ*^2^ değeri
		Positive(*n* = 48)	Negative(*n* = 26)		
IHA (Siemens) Total (*n* = 74)	Positive (*n* = 34)	32 (%)	2 (%)	< 0,001	23.62
	Negative (*n* = 37)	13 (%)	24 (%)		
	Intermediate (*n* = 3)	3 (%)	0		
IHA (Fumouse) Total (*n* = 74)	Positive (*n* = 35)	34 (%)	1 (%)	< 0,001	30.36
	Negative (*n* = 36)	11 (%)	25 (%)		
	Intermediate (*n* = 3)	3 (%)	0		
ELISA Total (*n* = 74)	Positive (*n* = 44)	41 (%)	3 (%)	< 0,001	38.19
	Negative (*n* = 29)	7 (%)	22 (%)		
	Intermediate (*n* = 1)	0	1 (%)		
IFA Total (*n* = 74)	Positive (*n* = 43)	40 (%)	3 (%)	< 0,001	35.71
	Negative (*n* = 31)	8 (%)	23 (%)		
	Intermediate (*n* = 0)	0	0		
WB Total (*n* = 74)	Positive (*n* = 38)	35 (%)	3 (%)	< 0,001	25.43
	Negative (*n* = 30)	10 (%)	20 (%)		
	Intermediate (*n* = 6)	3 (%)	3 (%)		

**Table 4:** Sensitivity, specificity, positive predictive value, negative predictive value and consistency values of different methods used in the detection of Anti-Echinococcus IgG

	IHA (Siemens)	IHA (Fumouse)	ELISA	IFA	WB
Sensitivity	66.67%	70.83%	85.42%	83.33%	72.92%
Specificity	92.31%	96.15%	88.46%	88.46%	88.46%
Positive predictive value	94.12%	97.14%	93.18%	93.02%	92.11%
Negative predictive value	60%	64.10%	76.67%	74.19%	63.89%
κ value (consistency)	0.53	0.60	0.71	0.69	0.56

## Discussion

Cystic echinococcosis is an endemic diseases in Türkiye [12, 13]. However, the apparent rates do not reflect the true prevalence of the disease, as the majority of data on the prevalence of these diseases is derived from hospital records and research conducted for academic purposes [6]. Therefore, the diagnostic techniques used to identify CE are critical and must be interpreted with extreme care.

Although a significant proportion of our patients reside in Ankara, the capital city of Türkiye, the precise city where the infected person acquired the parasite could not be determined due to the prolonged disease incubation period. Therefore, it is not possible to determine the exact exposure time to the parasite. Nevertheless, it should not be overlooked that there is a significant migration from rural to urban areas in the eastern provinces, where echinococcosis is common [13].

Currently, there are various methods for the diagnosis of CE in humans, but there is no accepted universal gold standard diagnostic method [14]. The diagnosis of CE is mostly based on clinical findings, imaging techniques, and serology [15].

IHA, ELISA IFA, and WB test are currently available from commercial companies for the diagnosis of CE. However, there may be some difficulties in interpreting the test methods that are used. In some patients, antibodies do not develop due to the size, location, structure, vitality of the cyst, and the inability of the patient’s immune system to be adequately activated and may cause false negativity. In addition, many factors such as test procedure and the properties of the antigen used in a test cause false negativities and false positives. This may cause imaging methods and serologic tests to be incompatible [16, 17].

Serological tests including indirect hemagglutination, ELISA, and latex agglutination tests usually have limited sensitivity and specificity due to antibody cross-reactivity in patients with certain disorders such as cirrhosis, malignancy, and other parasitic diseases. Experts in WHO consensus (in the WHO-Informal Working Group on Echinococcosis) recommended that specific serum antibodies should be evaluated using high-sensitivity serological tests and in dubious cases, they must be confirmed using a serological test with higher specificity [15].

The frequency of CE is not significantly different between men and women [1, 18]. In many studies, no significant relationship was found in terms of sex as a result of statistical calculations [19–22]. Several authors reported significant difference between the genders was found [23–26]. In these studies, the positivity of CE was found higher in women. It is thought that this is because women doing more of such work than men like cooking, cleaning, and collecting manure for fuel, as well as taking care and cleaning of dogs. Close contact with the definitive host and other environments where the eggs could be found are factors that increase the risk of contracting CE [24, 27]. In our study, the presence of CE was found to be significantly higher in female than in male, too (*p* < 0.05).

Cystic echinococcosis can occur in people of all ages. Different results have been reported about the distribution of the cases by age groups. In two studies in which cases in Malatya and its surroundings were investigated at different times, it was reported that patients with CE were mostly aged between 20 and 39 years [24, 28]. In a study conducted by Miman et al., with data from the province of Afyon, it was shown that positive cases were concentrated between the ages of 10–50 years, and peaked in the third decade [27]. In a retrospective study based on 22-year data in Diyarbakır, Kılınç et al., reported that the most common age range of patients was 20–30 years [29]. As a result of two separate studies conducted in the Public Health Institution of Türkiye and Konya, positive cases were found most frequently between the ages of 40–59, and their rates were 43.4% and 45%, respectively [30, 31]. In our study, the highest seropositivity (37.8%) was seen in patients aged 3–86 years, in the 40–59 age group, but this age range was not statistically significant. The presence of CE was found to be significantly higher in the 19–39 age group. In addition, it was found to be significantly lower in the 60–89 age group. We think that with age, the possibility of cyst formation due to reasons other than CE in the body increases, and as we age, the relative decrease in immunity and decrease in antibody production. Our findings are compatible with other studies conducted in our country.

It is known that the liver (65–70%) and lung (20%) are the most commonly affected organs of CE. Cases of CE that are localized in other organs such as the spleen, kidney, brain, bone, and heart are rare [4, 8]. Miman et al. reported that 53.8% of CE cases were located in the liver, 19.8% in the lung, 7.7% in the intraabdominal cavity, and 7.7% in other organs [27]. In another study conducted in Ankara, it was reported that CE was seen in the liver with a rate of 68.2%, followed by 17.4% in the liver and lung, 13% in the lung, and 4.4% in the spleen [32]. In a similar study conducted by Ertabaklar et al., it was reported that 81.8% of CE were in the liver, 6.1% in the lung and liver, 6.1% in the lung only, and lower rates in other organs (e.g. spleen, kidney, psoas) [33]. In our study, 74 of the seven patients (9.5%) had multiple localizations; six were in liver and lung, one was in the liver and intraabdominal cavity. Accordingly, it is seen that our results are compatible with other studies. Although not statistically significant, most cases (58/74) had cysts located in the liver.

Seropositivity rates in patients with a cross reactivity presumptive diagnosis of CE were found between 14 and 85% in many studies in our country [19, 20, 23–25, 34–38]. In our study, two different commercial IHA kits, ELISA, IFA, and WB tests were used to diagnoser CE seropositivity; 44.6% (33/74) were found positive with the Siemens IHA, 47.3% (35/75) with the Fumouze IHA, 58.1% (43/74) with ELISA, 56.7% (42/74) with IFA, and 51.3% (38/74) with the WB test. We think that the reason for the different rates of seropositivity is due to factors such as the characteristics and source of the antigens used in the tests, the immunity of the host, cyst localization, stage, and number.

It is known that IHA and ELISA tests are frequently used for the diagnosis of CE in routine clinical microbiology laboratories. In particular, IHA tests are preferred because they can be performed easily, results are obtained in a short time, do not require additional equipment, provide valuable information about antibody titres, and have high sensitivity [9, 10].

Akısu et al.‘s study included 31 patients with surgically proven lung CE, 18 patients with lung disease other than CE, and 10 healthy people, using IHA, ELISA, and WB tests. They determined the sensitivity of IHA, ELISA, and WB tests as 96.7%, 87.1%, and 100%, respectively, and the specificity was 82.2%, 89.2%, and 85.7%, respectively. In addition, false-positive results were detected in the WB test in four patients with lung disease other than lung CE [39]. In a study conducted by Deininger and Wellinghausen, a commercial line blot (LB) test in patients with suspected and proven CE was compared with a commercial WB test including the recombinant antigens (EgAgB, Em18, and Em95) that we used in our study. The researchers pointed out that the WB test might give false positive/negative results and there could be difficulty in distinguishing *E. granulosus* from *E. multilocularis* species [40].

In a study conducted by Doorn et al., 52 patients with confirmed CE, 237 patients with non-CE disease, and 10 healthy people were included. The Fumouze IHA test was used in the study, and the sensitivity and specificity of the test were found as 88% and 98.4%, respectively. In addition, one person from each group with neurocysticercosis, hookworm infection, filariasis, hepatic amoebiasis, malaria, toxoplasma, aspergillosis, HIV, and EBV infection was found to cross-react in the control group [41]. In the study of Sarı et al., 40 patients with confirmed CE and 40 controls were included. The sensitivity of the IHA, ELISA, and IFA tests was 90%, 87.5%, and 82.5%, and specificity was determined as 97.5%, 100%, and 100%, respectively. They found that a patient with taeniasis (2.5%) in the control group gave cross-reactivity [42].

Tamarrozzi et al. evaluated nine different commercial tests in patients who had hepatic CE and other focal liver lesions found by ultrasound. The tests were (5 ELISA, 2 WB, 1 immunochromatographic test, and 1 chemiluminescence immunoassay. The ELISA and WB tests used in their study had the same brand (Euroimmun) as ours. Among the tests, the specificity of Euroimmun WB and Euroimmun ELISA was the lowest. They also found the highest rate of intermediate results in the Euroimmun WB. They mentioned the difficulty of visually reading the WB test [43]. In our study, we found that the lowest specificity belonged to the ELISA, IFA, and WB tests. Similarly, we observed the highest doubtful result rate for WB. We obtained the WB test results with automatic reading but also had difficulty with visual reading, especially in intermediate cases.

In the study of Akgün et al., sera of 163 patients with preliminary diagnosis of CE were evaluated with IHA, ELISA and IFA tests. Western blot test was used to confirm the test results. The sensitivity of IHA, ELISA, and IFA tests were 88.76%, 78.65%, and 89.89%, and the specificity was 94.59%, 100% and 93.24%, respectively [19]. In a study of surgically confirmed or imaging-diagnosed CE patients, antibodies to Echinococcus in the serum of patients were investigated by IHA, ELISA, and WB tests. Sensitivity and specificity of IHA, ELISA and WB tests; 83.73%-73%, 80.72-87%, 81.33-99% were found, respectively [44]. In our study, the sensitivity of the Siemens IHA was 66.67%, specificity was 92.31%; the sensitivity of the Fumouze IHA was 70.83%, the specificity was is 96.15%; the sensitivity of ELISA was 85.42%, the specificity was 88.46%; the sensitivity and specificity of IFA were 83.33% and 88.46%; and the sensitivity and specificity of WB were 72.92% and 88.46%. The difference in sensitivity and specificity in tests is thought to be due to the difference in test methods, the characteristics of the antigen used in the test, the source of the antigen, and the patient’s antibody response [10, 42].

The sensitivity and specificity of the serological tests in patients with active cysts (CE1, CE2, and CE3) are generally higher than those with inactive-stage cysts such as CE4 and CE5. For these reasons, it is also very important to identify the cyst stage when evaluating the CE serological tests [43, 45, 46]. In our study, liver ultrasound examination reports indicated that 22 of the 74 patients had the WHO-IWGE stage classification. Among those 22 patients, 16 liver ultrasound reports belonged to the patients showing positivity in all serological tests whose cyst stages were defined as CE2-CE5. The remaining six reports belonged to the patients with inconsistencies between the serological tests, and their cyst stages were determined as CE1, CE4, or CE5.

In our study, ELISA (*κ* = 0.71) and IFA (*κ* = 0.69) were found to be the most consistent tests with pathology and radiology reports’ results. The test with the highest sensitivity was ELISA, and the second highest test was IFA. The specificity of two IHA tests were found higher than the other three tests. IFA cannot be included in the routine functioning of every laboratory due to the need for experience in the evaluation of IFA, as well as the need for a dark room and immunofluorescence microscope and the budget possibilities of the laboratories. For this reason, we suggest that IHA and ELISA tests at the minimum should be used together in the diagnosis of CE, considering the ease of application, less experience required, and spectrophotometers are widely available in routine laboratories. It has been stated that the WB can also be used as a diagnostic test in patients with negative results in routine tests but who are clinically and/or radiologically suspicious because of its high sensitivity and specificity [38]. However, it has been reported that WB can also give false positive/negative results. Therefore, confirmation tests that have higher sensitivity and specificity are needed [38, 40].

In conclusion, according to our findings, using a single method in the serologic diagnosis of CE is not sufficient. Using at least two methods together increases the diagnostic sensitivity and we suggest that these two tests could be IHA and ELISA for easy application in routine. Furthermore, when evaluating the results of the serological tests, examining the cyst’s stage is crucial. In addition, the lack of standardisation of the test results in CE is probably due to the distinct properties of the antigens utilised in serological tests. Also, this should be considered when assessing the results of serological tests.

## Limitations

In our study, cross-reactions against other diseases and parasites could not be evaluated. The other limitation is that we had no control group, and we could not access the reports showing the staging of CE cysts for all cases in the hospital records. In addition, the interpretation of serological test results may not be reliable in cases of immunodeficiency. We did not know the immune competence status of our patients.

## Abbreviations

IHA: Indirect Hemagglutination
ELISA: Enzyme-linked Immunosorbent Assay
IFA: Indirect Fluorescent Antibodies
WB: Western Blot
CE: Cystic Echinococcosis

## References

[1] McManus DP, Zhang W, Li J, Bartley PB (2003). Echinococcosis. Lancet (London England).

[2] Woolsey ID, Miller AL (2021). Echinococcus Granulosus Sensu Lato and Echinococcus multilocularis: a review. Res Vet Sci.

[3] Yıldız F (2019) Echinococcus Infection: The Effects of Echinococcosis on Public Health and Economy. International Journal of Veterinary and Animal Research (IJVAR), 2(2), 51–59. Retrieved from https://www.ijvar.org/index.php/ijvar/article/view/343

[4] Wen H, Vuitton L, Tuxun T, Li J, Vuitton DA, Zhang W, McManus DP (2019). Echinococcosis: advances in the 21st Century. Clin Microbiol Rev.

[5] WHO (19.02.2021). Echinococcosis. from https://www.who.int/news-room/fact-sheets/detail/echinococcosis

[6] Ok ÜZ, Kilimcioğlu AA, Özkol M (2020). Türkiye’de İnsanlarda Kistik Ekinokokkoz [Cystic echinococcosis in humans in Turkey]. Mikrobiyoloji Bulteni.

[7] Tamarozzi F, Akhan O, Cretu C. M, Vutova K, Akinci D, Chipeva R, Ciftci T, Constantin C. M, Fabiani M, Golemanov B, Janta D, Mihailescu P, Muhtarov M, Orsten S, Petrutescu M, Pezzotti P, Popa A. C, Popa L. G, Popa M. I, Velev V, Casulli A (2018). Prevalence of abdominal cystic echinococcosis in rural Bulgaria, Romania, and Turkey: a cross-sectional, ultrasound-based, population study from the HERACLES project.. The Lancet. Infectious diseases.

[8] Moro P, Schantz PM (2009). Echinococcosis: a review. Int J Infect Diseases: IJID: Official Publication Int Soc Infect Dis.

[9] Biava MF, Dao A, Fortier B (2001). Laboratory diagnosis of cystic hydatic disease. World J Surg.

[10] Siles-Lucas M, Casulli A, Conraths FJ, Müller N (2017) Laboratory diagnosis of Echinococcus spp. in human patients and infected animals. Advances in parasitology. 96:159–257. 10.1016/bs.apar.2016.09.003 10.1016/bs.apar.2016.09.003 28212789

[11] Landis JR, Koch GG (1977). The measurement of observer agreement for categorical data. Biometrics.

[12] Baş Y, Beyhan Y, Keser Şahin H, Özçerezci T, Karasartova D, Güreser A, Güney G, Taylan Ozkan A (2021) Türkiye Parazitoloji Dergisi 45(4):262–267. 10.4274/tpd.galenos.2021.22931. Çorum’da Histopatolojik Olarak Echinococcus Tanısı Almış, Formalinle Fikse Parafine Gömülü Doku Örneklerinin Değerlendirilmesi 10.4274/tpd.galenos.2021.22931 34889193

[13] Gurler A, Bölükbaş C, Acici M, Umur Ş (2019). Türkiye ve Dünya’da Echinococcus multilocularis’in Yayılışına Genel Bakış. Türkiye Parazitoloji Dergisi.

[14] Barnes TS, Deplazes P, Gottstein B, Jenkins DJ, Mathis A, Siles-Lucas M, Torgerson PR, Ziadinov I, Heath DD (2012). Challenges for diagnosis and control of cystic hydatid disease. Acta Trop.

[15] Brunetti E, Kern P, Vuitton DA, Writing Panel for the WHO-IWGE (2010). Expert consensus for the diagnosis and treatment of cystic and alveolar echinococcosis in humans. Acta Trop.

[16] Altıntaş N, Yazar S, Tınar R, Çoker A, Izmir (2004) Hidatoloji Derneği, 159–180

[17] Kilimcioğlu AA, Girginkardeşler N, Korkmaz M, Özkol M, Düzgün F, Östan I, Pabuşcu Y, Dinç G, Ok UZ (2013). A mass screening survey of cystic echinococcosis by ultrasonography, Western blotting, and ELISA among university students in Manisa. Turk Acta Trop.

[18] Kern P, Menezes da Silva A, Akhan O, Müllhaupt B, Vizcaychipi KA, Budke C, Vuitton DA (2017) The echinococcoses: diagnosis, Clinical Management and Burden of Disease. Advances in parasitology. 96:259–369. 10.1016/bs.apar.2016.09.006 10.1016/bs.apar.2016.09.006 28212790

[19] Akgün S, Sayi̇ner HS, Karsligi̇l T (2017). Kistik Ekinokokoz’un serolojik tanısında Indirekt Hemaglütinasyon, İndirekt Floresan Antikor ve Enzim İmmuno Assay testlerinin etkinliğinin değerlendirilmesi. J Contemp Med.

[20] Cengiz ZT, Yılmaz H, Beyhan YE, Kotan MÇ, Çobanoğlu U, Ekici A, Ödemiş N (2015) Turkiye parazitolojii dergisi 39(3):209–211. 10.5152/tpd.2015.3995. [Cystic Echinococcosis Seropositivity in the Blood Samples Sent to Parasitology Laboratory of Yüzüncü Yıl University Medical Faculty between 2005 and 2013: Retrospective Assessment] 10.5152/tpd.2015.3995 26470927

[21] Park SJ, Han SS, Anvarov K, Khajibaev A, Choi MH, Hong ST (2015). Prevalence of serum IgG antibodies to Cystic Echinococcus Antigen among patients in an Uzbekistan Emergency Hospital. Korean J Parasitol.

[22] Karaman U, Mıman O, Kara M, Gicik Y, Aycan OM, Atambay M (2005). Turkiye Parazitolojii Dergisi.

[23] Cetinkaya U, Hamamcı B, Kaya M, Gücüyetmez S, Kuk S, Yazar S, Sahin I (2012). [Investigation of anti-echinococcus granulosus antibodies in patients with suspected cystic echinococcosis]. Turkiye Parazitolojii Dergisi.

[24] Daldal Ü, Atambay M, Aycan T, Yıldız N, Kaya Ö (2012). [Evaluation of patients presenting with a suspicion of cystic echinococcosis to the Serology Laboratory of the Parasitology Department of Inonu University Medical Faculty]. Mustafa Kemal Üniversitesi Tıp Dergisi.

[25] Beyhan YE, Babür C, Mungan M, Özkan AT (2015). [Evaluation of cystic echinococcosis suspected patients Applied to National Parasitology Reference Laboratory of Public Health Institution of Turkey between 2009–2013]. Turkiye Parazitolojii Dergisi.

[26] Öztürk-Durmaz Ş, Kesimal U, Turan M (2020). [Evaluation of Cyst Hydatid cases: one Center’s experience over a two-year period]. Klimik Dergisi.

[27] Mıman O, Atambay M, Aydin NE, Daldal N (2010). [The clinical, serological and morphological analysis of 91 patients with cystic echinococcosis following surgery]. Turkiye Parazitolojii Dergisi.

[28] Karaman Ü, Daldal N, Atambay M, Aycan Ö (2002). Serological results of cases with a presumptive diagnosis of Hydatid Cyst during 1999–2002 in the İnönü University Medical Faculty, Malatya. İnönü Üniversitesi Tıp Fakültesi Dergisi.

[29] Kılınç N, Uzunlar A, Özaydın M (2003). [Uncommonly localized cases of echinococcosis (report of 45 cases)]. Türkiye Ekopatoloji Dergisi.

[30] Kılıç S, Babür C, Taylan Özkan A (2007). [Comparison of the results of Indirect Hemagglutination and ELISA methods for the cases prediagnosed as Hydatid Cyst Disease]. Mikrobiyoloji bülteni.

[31] Uysal E, Ozdemir M, Baykan M (2009). [Comparison of Commercial IFA, IHA and In-house IFA tests in the diagnosis of cystic Echinococosis]. Turkiye Parazitol Derg.

[32] Eşgin M, Aktaş M, Coşkun Ş (2007). [The investigation of antibody Presence in the Sera of patients with a suspicion of cystic echinococcosis by using Indirect Hemaglutination Test (IHA)]. Turkiye Parazitolojii Dergisi.

[33] Ertabaklar H, Yıldız İ, Malatyalı E, Tileklioğlu E, Çalışkan SÖ, Ertuğ S (2019). Retrospective analysis of cystic echinococcosis results in Aydın Adnan Menderes University Training and Research Hospital Parasitology Laboratory between 2005 and 2017. Turkiye Parazitolojii Dergisi.

[34] Delibaş-Bayram S, Özkoç S, Şahin S, Aksoy Ü, Akısü Ç (2006) [Evaluation of patients presenting with a suspicion of cystic echinococcosis to the Serology Laboratory of the Parasitology Department of Dokuz Eylül University Medical Faculty]. Turkiye parazitolojii dergisi, 30, 279– 81 17309027

[35] Karadağ A, Yanık K, Ünal N, Odabaşı H, Hökelek M (2013). [Evaluation of materials sent due to suspected cystic echinococcosis to the parasitology laboratory of Ondokuz Mayıs University Medical School between the years 2005–2011]. Turkiye Parazitolojii Dergisi.

[36] Çitil B, Tunçoğlu E, Erbil Ö, Değirmenci M, Özenoğlu A, Sert H (2014). Adıyaman’Da Kistik Ekinokokkozis Ön Tanılı Hastaların İndirekt Hemaglütinasyon (İHA) Yöntemi Ile Değerlendirilmesi. Van Tıp Derg.

[37] Aslan M, Kurt A, Vural M (2019). [The investigation of Indirect Hemaglutination (IHA) Test results of patient with early diagnosis cystic echinococcosis]. Van Tıp Dergisi.

[38] Güreser A, Duman G, Sarzhanov F, Karasartova D, Dogruman-Al F, Taylan-Ozkan A (2019). Western blot assay of Anti-echinococcus granulosus antibody positive serum samples by Indirect Haemagglutination Method. Türk Hijyen Ve Deneysel Biyoloji Dergisi.

[39] Akisu C, Delibaş B, Yuncu G, Aksoy U, Ozkoç S, Biçmen C (2005). [Evaluation of IHA, ELISA and Western blot tests in diagnosis of pulmonary cystic hidatidosis]. Tuberkuloz ve Toraks.

[40] Deininger S, Wellinghausen N (2019). Evaluation of a new combined western and line blot assay (EUROLINE-WB) for diagnosis and species identification of Echinococcus infection in humans. GMS Infect Dis.

[41] van Doorn HR, Hofwegen H, Koelewijn R, Gilis H, Wentink-Bonnema E, Pinelli E, van Genderen PJ, Schipper HG, van Gool T (2007). Reliable serodiagnosis of imported cystic echinococcosis with a commercial indirect hemagglutination assay. Diagn Microbiol Infect Dis.

[42] Sari C, Ertuğ S, Karadam S, Özgün H, Karaoğlu A, Ertabaklar H (2009). [The comparative evaluation of Enzym Lynked Immunosorbent Assay (ELISA), Indirect Hemagglutination Test (IHA) ve Indirect fluorescent antibody test (IFAT) in the diagnosis of cystic Echinococcosis]. Turkiye Parazitol Derg.

[43] Tamarozzi F, Longoni SS, Vola A, Degani M, Tais S, Rizzi E, Prato M, Scarso S, Silva R, Brunetti E, Bisoffi Z, Perandin F (2021). Evaluation of nine commercial serological tests for the diagnosis of human hepatic cyst echinococcosis and the Differential diagnosis with other Focal Liver lesions: a diagnostic accuracy study. Diagnostics (Basel Switzerland).

[44] Zait H, Hamrioui B (2020). Human cystic echinococcosis: serological diagnosis by indirect hemagglutination test, enzyme-linked immunosorbent assay, immunoelectrophoresis, and immunoblotting in surgically confirmed patients versus cases diagnosed by imaging techniques. Med et maladies Infectieuses.

[45] Tamarozzi F, Silva R, Fittipaldo VA, Buonfrate D, Gottstein B, Siles-Lucas M (2021). Serology for the diagnosis of human hepatic cystic echinococcosis and its relation with cyst staging: a systematic review of the literature with meta-analysis. PLoS Negl Trop Dis.

[46] Lissandrin R, Tamarozzi F, Piccoli L, Tinelli C, De Silvestri A, Mariconti M, Meroni V, Genco F, Brunetti E (2016). Factors influencing the Serological response in hepatic Echinococcus granulosus infection. Am J Trop Med Hyg.

